# Service Prof. E. Andrews, Surgeon-in-Charge

**Published:** 1862-03

**Authors:** 


### The Clinique.

### MERCY HOSPITAL—SURGICAL WARDS.

**Service Prof. E. ANDREWS, Surgeon-in-Charge.**

### *Reproduction of the Inferior Maxillary—Preservation of the Teeth.*

Reported for the EXAMINER.

GENTLEMEN:—We have here an unusual case. A few weeks ago, I removed from this man nearly the whole of the right half of the lower jaw, yet, singular to relate, I have saved his teeth, which are now growing firm in the new bone which is replacing the old. The only instance like it which I ever heard of, is related in *Championniere's Journal* in the following words:—

"In this case the right side of the inferior maxillary was extracted *in toto*, with the articular condyle, and has been so perfectly replaced by the efforts of nature, that is now almost impossible to discover which side of the jaw was removed by the surgeon. A singular peculiarity is the preservation of the teeth, which were left by the operator, attached to the gums as moveable as a string of beads, and became subsequently consolidated by the closing up of the ossified layers secreted by the periosteum. Unfortunately, after having performed their functions for the space of two years, they one after another dropped out, but the patient nevertheless manages to masticate his food, and to speak distinctly."

In the case before us, the disease commenced some three months after a contusion of the head, and progressed until it necrosed the right half of the jaw from the symphysis back to the neck of the bone, the condyle escaping injury. In this condition he was brought to me. I made an incision along the base of the jaw, and seizing the dead bone with the forceps, drew it readily out. On examination, I was surprised to perceive that the sockets of the teeth were empty, they having remained attached to the gum, and slipping easily from their cavities when the bone was drawn down. The finger introduced into the wound felt the row of fangs, apparently clothed with periosteum and strongly attached to the gums, but easily moved about. In some parts of the cavity, the periosteum had begun to reproduce a shell of bone, and taking courage from this indication, I determined to leave the teeth in position, in hope that the new growth of bone would enclose the fangs, and thus render the teeth firm and useful. The result has justified my expectations. You see that the arch of the jaw is becoming firm, and the teeth instead of hanging loose in the gum, are set strongly, giving the sensation to the finger as though they were set in cartilage, or other semi-indurated tissue. Whether they will drop out after two or three years' service, as in the case I quoted you, remains to be seen. Since the removal of the jaw proves the loss of the dental artery, nerve and vein, it becomes necessary to explain in what manner the teeth obtain nutrition after the necrosis of the jaw. The teeth are not set in solid bone; on the contrary, there is a periosteum lining the socket, and intervening between the fang and the bone. When necrosis of the jaw occurs, this periosteal lining, like the similar membrane on the outside of the bone, may retain its vitality, and supply to the dental artery, by inosculation, the blood which is no longer transmitted through the dental canal. The absorbent action of the membrane upon the bone enlarges the space between the fang and the socket, and thus loosens the tooth, so that, at the time of operation, it readily slips from its bed, and the necrosed bone is drawn from it, as a boot is drawn from a foot.

Many remarkable instances are on record of complete restoration of the jaw after removal for necrosis, but these two are the only cases which I know of, where the teeth were preserved, so as to have a living attachment to the new bone.

You will find in practice, that the inferior maxilla is more likely to be reproduced after necrosis than any other bone in the body; hence, calculations should always be made on this event, and the following directions be observed:—

First, the operation should not be performed early in the disease, unless there is some spreading disease, as epulis, which requires to be limited by excision. In cases of necrosis, which involve a large portion of the jaw at once, with little danger of subsequent extension, it is important to preserve the sequestrum in position until it is fully separated from the living bone, and if the irritation is not too great, until the new shell of bone produced from the periosteum has attained solidity enough to maintain the form of its arch.

Secondly, before removing the bone, care should be taken to ascertain whether the teeth implanted in it are loose, and if so, to prevent their being torn from the gum with the bone. By a little care, the bone may be slipped downward, and the teeth left in position.

Finally, if the arch of new ossific matter be not sufficiently consolidated to maintain its form alone, art may often supply the weakness of nature, The cases of death of both halves of the maxilla are comparatively rare; and, hence in ordinary cases, the sound side may be made to support the weak one. Before the operation of removal is performed, a dentist should be called on to take a mould of both upper and lower teeth on the sound side. By this method, he can prepare accurately-fitting gold plates to cap the rows of teeth and the gums. The lower plate should be soldered or riveted to the upper in the natural position, so that when placed in the mouth, the arch of the lower teeth will be secured by the support of the former. After the operation, if the new bone lacks strength, the chin will be found to fall over towards the affected side, but the insertion of the plate and the application of a bandage, pressing the chin upward, will hold the lower maxilla accurately in position, until the consolidation of the new bone renders artificial support unnecessary.

Silver plate will do equally well, so far as mere mechanical strength is concerned, but it has the disadvantage of immediately turning black, aud presenting a repulsive appearance to the friends of the patient. If a cheaper article than gold is required, probably the prepared gutta percha, recently introduced for the plates of artificial teeth, would be found available. The preservation of the correct line of the chin is of great importance in the female sex, because any deformity in them, cannot be concealed by a beard; hence, you will exert yourself in such cases to obtain the most perfect of possible results. I have seen bungling efforts to keep the correct position, by wiring one or two of the upper and lower teeth together. But all such attempts are unskilful. The support thus obtained, is not only precarious, but temporary; for where the whole strain is brought to bear on one tooth, the absorption of the alveolar process under the pressure, loosens and displaces the tooth, and often causes its speedy loss; but a pressure which is distributed over all the teeth of one side, is well borne, and may be long maintained. Similar mechanical devices are often the best possible splints for fractures of the inferior maxilla, particularly where the bone is broken into several fragments.

In the surgery of this bone, gentlemen, your ingenuity must be prompt, patient, and fertile. Do not be discouraged by disheartening appearances. By faithfulness and skill, the face of your diseased or injured patient will at length assume a natural aspect, and you will obtain a success which often exceeds your most sanguine expectations.

---

Potassio-tartrate of iron in Rheumatism.—Rheumatism is one of those affections which at times resist all the ordinary modes of treatment in use, though occasionally it will readily yield to a remedy not commonly employed in that affection. This result we have observed in a case under Dr. Willshire's care, in the person of a somewhat pallid girl, with rheumatism of an erratic character, the pains flying about, as it were, to various parts of the body. To this she had been subject for the last three years, and nothing seemed to produce any marked improvement; she was, therefore, ordered a mixture containing the potassio-tartrate of iron, which has produced a decidedly beneficial effect in a short period of time, the pains having become gradually diminished, but not as yet wholly gone, and her health is better. In obstinate and suitable cases, this excellent preparation of iron should be borne in mind.—*Lancet.*

